# Lineage shift in Indian strains of Dengue virus serotype-3 (Genotype III), evidenced by detection of lineage IV strains in clinical cases from Kerala

**DOI:** 10.1186/1743-422X-10-37

**Published:** 2013-01-29

**Authors:** Anoop Manakkadan, Iype Joseph, Raji Rajendran Prasanna, Riaz Ismail kunju, Lalitha Kailas, Easwaran Sreekumar

**Affiliations:** 1 Viral Disease Biology Program, Rajiv Gandhi Centre for Biotechnology (RGCB), Thycaud P.O, Thiruvananthapuram, 695014, Kerala, India; 2 Department of Pediatrics, Sree Avitom Thirunaal (SAT) Hospital, Government Medical College, Thiruvananthapuram, Kerala, India; 3 Community Health Centre (CHC), Department of Health Services, Pathanapuram, Kollam, Kerala, India

**Keywords:** Dengue virus, Serotype 3, Viral diversity, Evolution, Lineage turn-over

## Abstract

**Background:**

Local epidemiology of Dengue is defined by the genetic diversity of the circulating Dengue virus (DENV) strains. This important information is not available for the virus strains from most parts of the Indian subcontinent. The present study focused on the genetic diversity of the serotype 3 DENV strains (DENV-3) from India.

**Results:**

A total of 22 DENV-3 strains identified by reverse-transcription PCR analysis of serum samples from 709 patients were studied. These samples were collected over a period of 4 years (2008–2011) from dengue fever suspected patients from Kerala, a dengue endemic state in South India. Comparison of a 1740bp nucleotide sequence of the viral Capsid-Pre-membrane-Envelope coding region of our strains and previously reported DENV-3 strains from India, South Asia and South America revealed non-synonymous substitutions that were genotype III-specific as well as sporadic. Evidence of positive selection was detected in the I81 amino acid residue of the envelope protein. Out of the 22 samples, three had I81A and 18 had I81V substitutions. In the phylogenetic analysis by maximum likelihood method the strains from Kerala clustered in two different lineages (lineage III and IV) within genotype III clade of DENV-3 strains. The ten strains that belonged to lineage IV had a signature amino acid substitution T219A in the envelope protein. Interestingly, all these strains were found to be closely related to a Singapore strain GU370053 isolated in 2007.

**Conclusions:**

Our study identifies for the first time the presence of lineage IV strains in the Indian subcontinent. Results indicate the possibility of a recent exotic introduction and also a shift from the existing lineage III strains to lineage IV. Lineage shifts in DENV-3 strains have been attributed to dramatic increase in disease severity in many parts of the world. Hence the present observation could be significant in terms of the clinical severity of future dengue cases in the region.

## Background

Dengue, a mosquito vector transmitted viral infection, has emerged as a major disease in recent times [1,2]. It is prevalent in most of the tropical regions and affects around 100 million people annually. Over the years, there is an exponential increase in the cases of Dengue fever (DF) or its severe forms, Dengue hemorrhagic fever (DHF)/Dengue shock syndrome (DSS) across the world. This augmented incidence of dengue has been attributed to increased air-travel, increased urbanization, amplified mosquito population due to deterioration in the public health infrastructure and changing climatic conditions [2].

The Dengue virus (DENV) is a positive-stranded RNA virus of the *Flaviviridae* family consisting of 4 serotypes (DENV-1, 2, 3, and 4). Infection with one serotype confers life-long protective immunity against a future infection with the same serotype, but not against the other three [2,3]. The ~11,000 base-pairs (bp) long viral genome consists of a small 5’non-translated region (5’NTR) of ~100 bp; a 10179 bp open reading frame that codes for the three structural and seven non-structural proteins; and a 3’NTR of ~400 bp [2]. The Capsid (C), Membrane (M) and Envelope (E) proteins form the structural proteins and NS1, NS2A, NS2B, NS3, NS4A, NS4B and NS5 proteins form the non-structural proteins. The NS5 protein functions as the viral RNA-dependent RNA polymerase that is responsible for replicating the genome [4]. This protein has a low fidelity during the viral genome replication contributing to the genomic variability within the viral serotypes and a constantly changing epidemiology of dengue. Identifying such changes by genome analysis has become a major tool in understanding dengue disease dynamics [5]. Previous studies have reported that almost all gene segments of the virus are useful in generating information on the viral evolution [6,7], though whole genome analysis has gained more emphasis recently [8-10].

Dengue virus has been prevalent in the Indian subcontinent for the last 50 years [11]. Like Southeast Asia, the region has become hyperendemic to dengue with the circulation of all the four serotypes [12]. Reflecting the global trend, the disease incidence has been increasing with more cases of DF and DHF over the years exhibiting a change in disease epidemiology [13]. DENV-1 and DENV-3 have been reported to be associated with increased incidence of mortality in the country [14]. In the published literature, genetic studies on the dengue virus from India have mostly used strains from Northern and Western parts of the subcontinent [7,10,14,15]. The southern region, however, experiences regular incidences of small-scale outbreaks of dengue [16-19], but there has been a very limited study to understand the serotypes and genotypes of the virus prevalent in this region. Since its first documentation in Kerala 44 years ago, dengue remained a low profile disease in the state till its re-emergence in epidemic form causing significant morbidity and mortality in 2003 [16]. As in other parts of the country, the dengue virus activity in the state has gone up considerably in recent years [11]. Our earlier studies have identified dengue outbreaks in Kerala that had significant number of patients with concurrent infections involving multiple serotypes and also the circulation of DENV-1 strains that are possibly introduced from neighboring countries [18,19]. A progressive molecular change in the viral strains, especially DENV-1, was also observed [19].

The replacement of DENV-1 with DENV-3 in the dengue incidences in Northern India has been reported recently indicating viral serotype swings in the region [20]. The serotype and genotype shifts occurring in circulating dengue viral strains in a locality are important causes for enhanced severity of dengue outbreaks [13,21-23]. At times, even the more subtle changes such as the ones at the lineage level could alter the potential of the dengue virus to cause severe disease. This has been observed in Sri Lankan DENV-3 strains [24-26]. In the present study, we characterized DENV-3 strains circulating in Kerala from 2008–2011 and compared the data with other Indian and global strains to understand the genetic variations. The information generated would be important in deciphering dengue in the region at a molecular level.

## Results

### Clinical samples, viral nucleic acid and anti-dengue IgG detection

**Figure 1:**
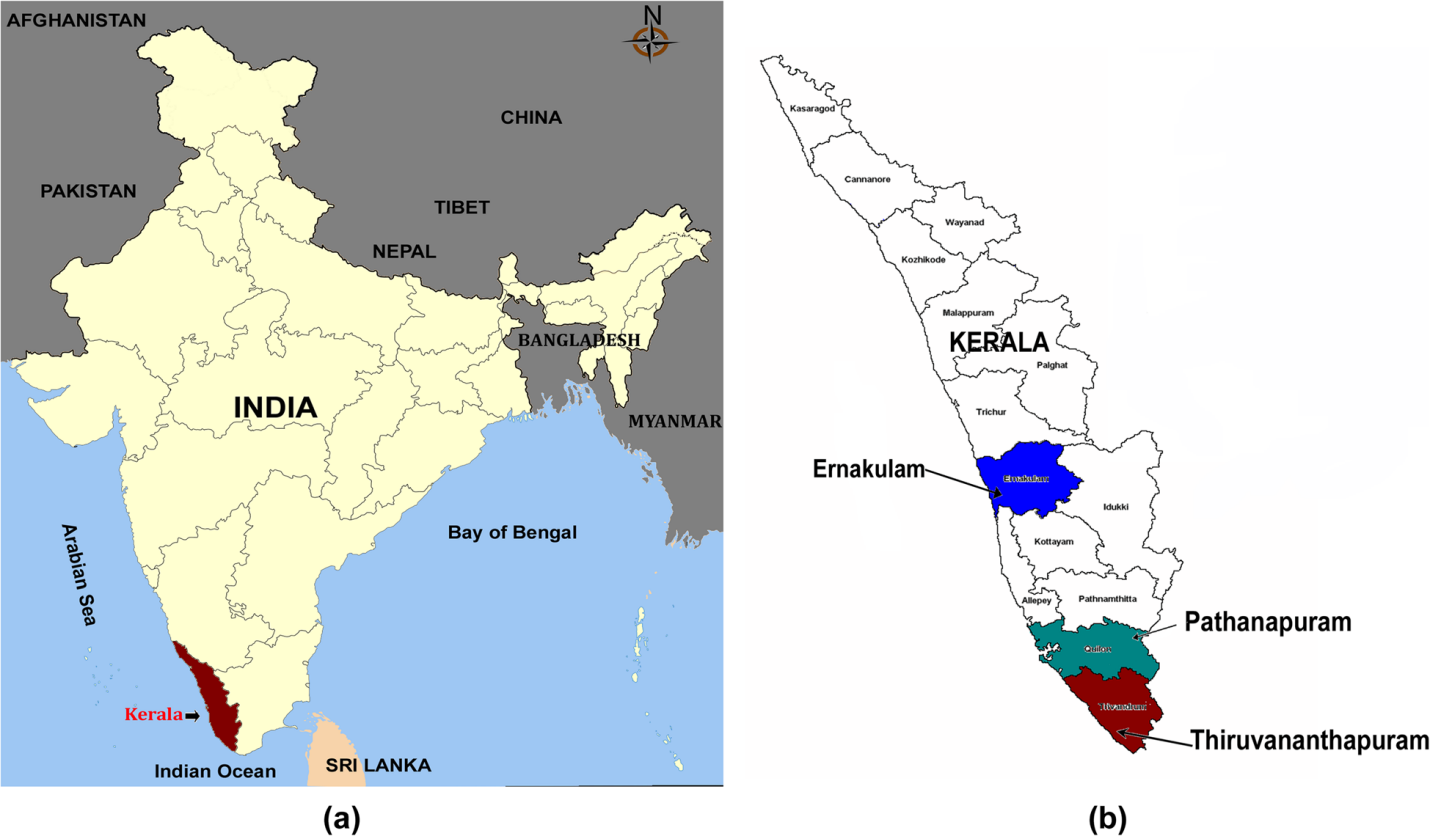
**Maps of (a) India and (b) Kerala. **The sites of sample collection for the study are shown.

In the four year period (2008–2011) of the study, acute-phase serum samples from dengue-suspected patients were collected from different parts of Kerala (Figure 1). A total of 709 samples were analyzed by RT-PCR. Among them, 162 samples were positive for viral nucleic acid (22.84%), and by RT-PCR-based typing 56 were DENV-1 (7.9%), 29 were DENV-2 (4%) and 32 were DENV-3 (4.5%). DENV-4 strains could not be identified in the clinical samples obtained during this period. Some of these samples showed co-infection with multiple serotypes [18]. Characterization of the DENV-1 strains from these samples has been reported earlier [19]. In the present study, we focused on the DENV-3 strains in the samples that were obtained from single serotype infections (n=22) (Table 1). Anti-dengue IgG ELISA carried out in these samples to detect pre-existing immunity found 11 samples to be positive. 

**Table 1:** Details of samples used in the study

**Sl no.**	**Strain name**	**Age /Sex**	**Date of collection**	**Place of collection**	**GenBank accession number**
1	RGCB350/08	7/M	19-05-2008	Thiruvananthapuram	JX070113
2	RGCB353 /08	9/M	29-05-2008	Thiruvananthapuram	JX070114
3	RGCB429/08	20/M	28-11-2008	Ernakulam	JX070115
4	RGCB544/09	6Mnth/M	09-01-2009	Ernakulam	JX070116
5	RGCB632/09	30/M	25-04-2009	Thiruvananthapuram	JX070117
6	RGCB655/09	17/F	07-05-2009	Thiruvananthapuram	JX070118
7	RGCB804/10	5/F	16-03-2010	Thiruvananthapuram	JX070119
8	RGCB815/10	12/F	23-05-2010	Pathanapuram	JX070120
9	RGCB825/10	22/M	04-06-2010	Pathanapuram	JX070121
10	RGCB828/10	24/F	05-06-2010	Pathanapuram	JX070122
11	RGCB830/10	33/M	08-06-2010	Pathanapuram	JX070123
12	RGCB834/10	26/F	12-06-2010	Pathanapuram	JX070124
13	RGCB837/10	42/F	14-06-2010	Pathanapuram	JX070125
14	RGCB838/10	45/F	14-06-2010	Pathanapuram	JX070126
15	RGCB915/10	28/M	21-12-2010	Thiruvananthapuram	JX070127
16	RGCB988/11	10/M	07-04-2011	Thiruvananthapuram	JX070128
17	RGCB1037/11	38/M	10-06-2011	Thiruvananthapuram	JX070129
18	RGCB1200/11	55/M	20-12-2011	Thiruvananthapuram	JX070130
19	RGCB1201/11	50/F	20-12-2011	Thiruvananthapuram	JX070131
20	RGCB1205/11	28/F	23-12-2011	Thiruvananthapuram	JX070132
21	RGCB1270/11	20/M	01-03-2011	Thiruvananthapuram	JX070133
22	RGCB1271/11	15/F	10-06-2011	Thiruvananthapuram	JX070134

### Detection of DENV3 infection of C6/36 cells by immunofluorescence

**Figure 2:**
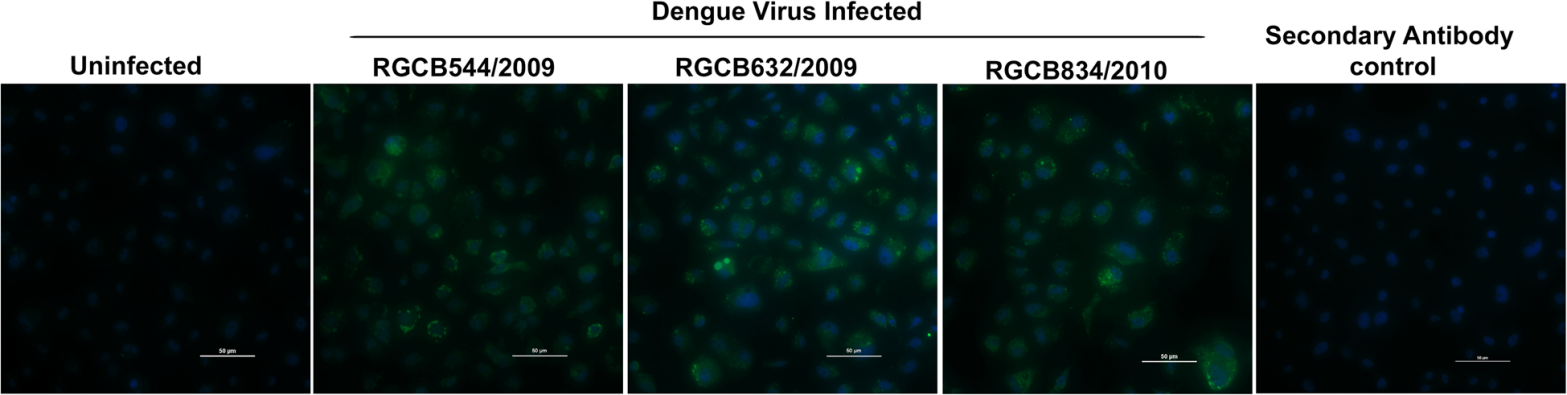
**Immunofluorescence analysis of DENV-3 infected C6/36 mosquito cell line. **Cells grown in 24-well cell culture plates were infected with the indicated virus strains for three days. The viral replication was detected by immunostaining using anti-dengue virus polyclonal serum as primary antibody and goat anti-rabbit IgG Alexa Fluor® 488 conjugated antibody as the secondary antibody. Uninfected cells and cells treated only with the secondary antibody are shown as controls. The cytoplasmic fluorescent foci in the infected cells indicate the sites of virus replication. Blue staining regions indicate the nuclei of the cells counterstained with DAPI . Scale bar represents 50 μm.

Immunofluorescence analysis of infected cells indicated presence of viral replication as evidenced by discrete cytoplasmic fluorescence foci. Control cells did not show any fluorescence signal in the analysis (Figure 2). Further, the presence of virus was confirmed by RT-PCR amplification of the RNA isolated from the supernatant of infected cultures (data not shown). 

### Sequence analysis of the C-PrM-E region

**Figure 3:**
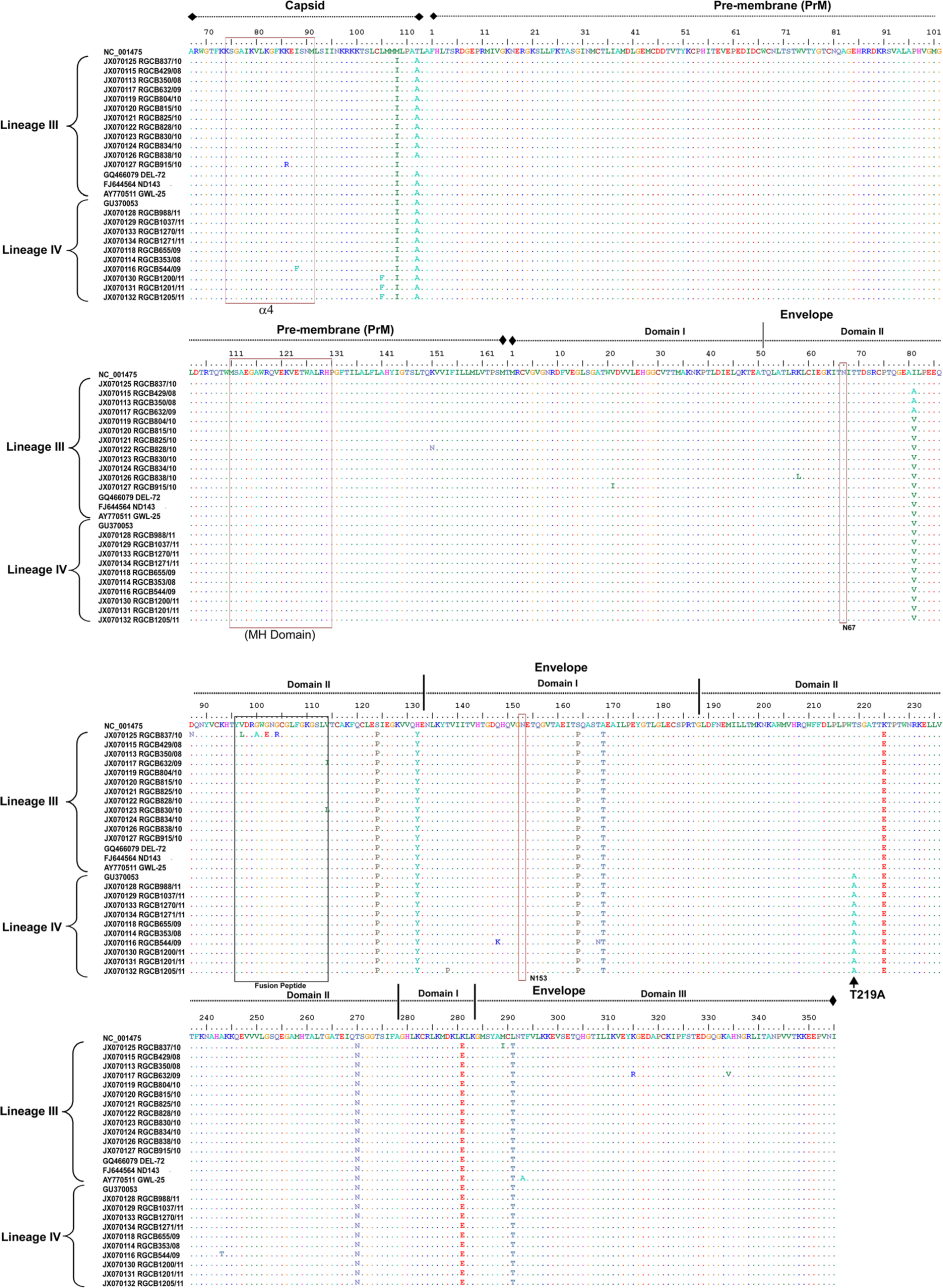
**Comparative amino acid sequence analysis of the Capsid-PrM-Envelope region of DENV-3 strains. **The amino acid sequences derived from the nucleotide sequences of the capsid, pre-membrane and envelope coding region of the NCBI reference strain (NC_001475), viral strains from Kerala and other closely related Indo-Pacific strains were aligned using Clustal W and compared. The domains and structural features were located in the aligned sequences based on the previous report [31] and they are indicated in the figure. The signature amino acid substitution T219A in lineage IV strains newly identified in the study is shown with an arrow.

A 1740 bp sequence (Figure 3; from position 291 to 2030 with respect to the NCBI DENV-3 reference sequence NC_001475) was used in the analysis. The sequence corresponds to the coding region for a partial Core protein, complete PrM region and partial envelope protein. The region of the 22 sequences analyzed (Table 1) had an average 94.4% nucleotide identity with the (NC_001475) reference strain. Identity comparison with the sequences of the closely related Indian strains GWL-25 (GenBank Accession No.AY770511), ND-143 (FJ644564) and DEL-72(GQ466079), which have been studied by whole genome sequencing [10], two groups were detected - 11 sequences with an average identity of 96.9% and remaining 11 sequences with an average identity of 99.1%. These two groups showed a clearly distinct nucleotide substitution pattern spanning across the 1740 bp region. Comparison of the translated amino acid sequence of the region also showed this delineation and revealed several substitutions (Figure 3). Some of these substitutions, such as the M108I, T112A in the capsid and I81A/V, S124P, H132Y, S164P, A169T, K225E, T270N, K281E and L291T in the envelope protein, were genotype specific substitutions present in all the sequences compared (Figure 3). Three strains (RGCB1200/11; RGCB1201/11 and RGCB1205/11) had an L115F change in the capsid protein and ten strains had T219A mutation in the envelope protein. 

### Phylogenetic analysis

**Figure 4:**
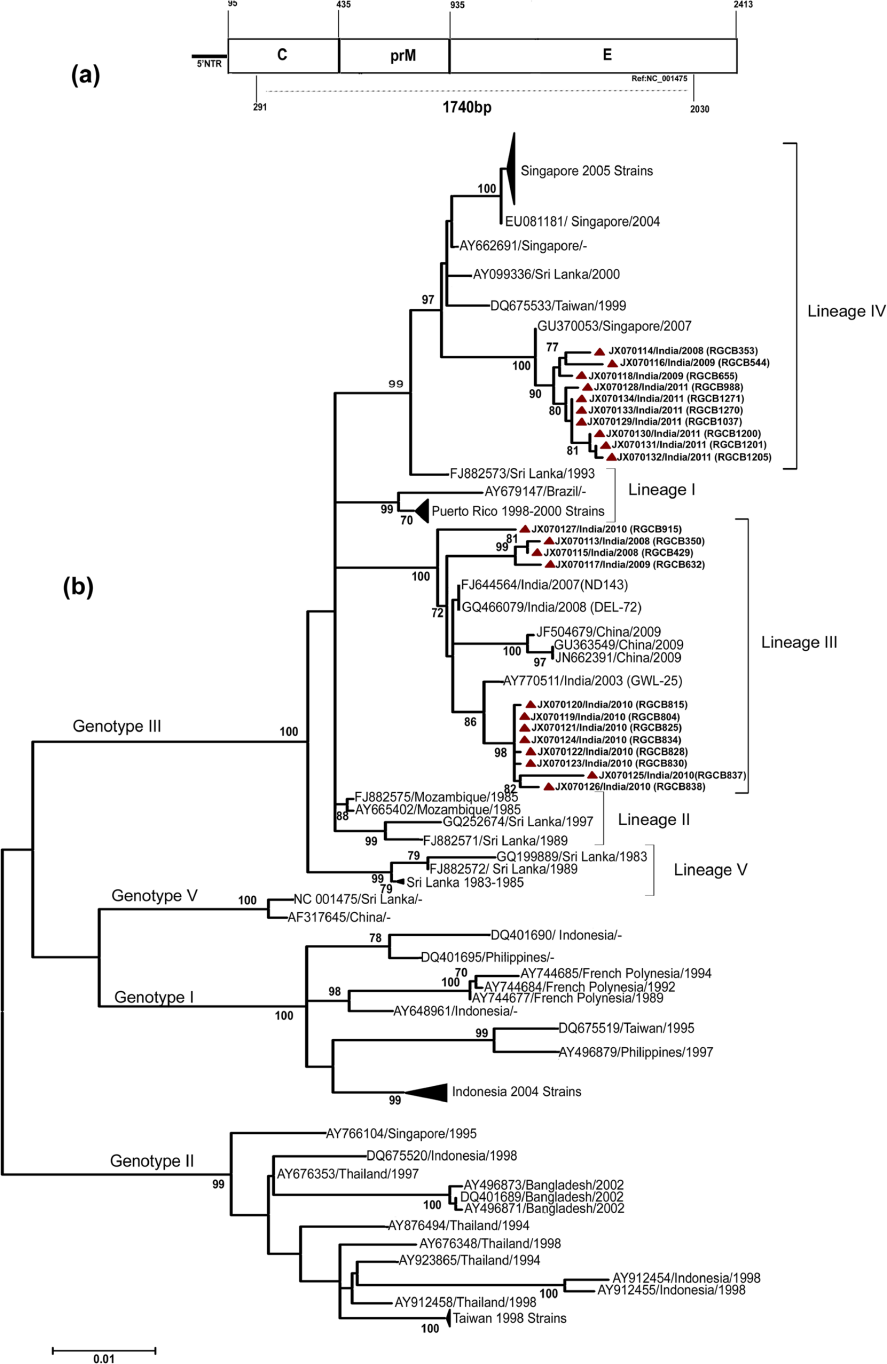
**a. The Capsid-PrM-Envelope sequence region used in phylogeneticAnalysis. **The positions are numbered with reference to the NCBI strain NC_001475. **b**. Phylogenetic analysis using Maximum-Likelihood method. Nucleotide sequences of a 1740 bp partial C-PrM-E coding region from 158 dengue serotype-3 virus representing the Indian, Asian and South American strains were used in the analysis. Analysis was carried out with 1000 bootstrap replications employing a Tamurai-Nei substitution model with Gamma distributed (G) rate among sites. The scale bar represents the number of nucleotide substitutions per site. Boot–strap values more than 70% are shown. GenBank accession number, place and year of isolation are indicated. Sequences obtained in the study are shown with a filled triangle (‘▲’). To improve clarity, the clades representing the related strains from Singapore, Puerto-Rico, Sri Lanka, Indonesia and Taiwan are shown compressed.

The 1740 bp Capsid-Pre-membrane-Envelope (C-PrM-E) coding region (Figure 4) of 158 sequences representing four major genotypes of the DENV-3 (genotype I, II, III, V) was used in the maximum likelihood analysis employing a Tamurai-Nei substitution model with Gamma distributed (G) rate among sites. A clear delineation of the four genotypes was evident in the phylogram (Figure 4). All the Indian strains used in the analysis, including the ones from the present study, clustered within the genotype III. In agreement with previous reports [10,14,20], five lineages were decipherable within the genotype III clade, which had a significant boot-strap support (>70%). The isolates from Kerala were distributed between the lineage III and lineage IV, whereas the three Indian strains used in the analysis clustered within the lineage III. The lineage III strains from Kerala were closely related to the GWL-25 (AY770511), ND143 (FJ644564) and DEL-72 (GQ466079) strains from India used in the analysis. The lineage IV strains from Kerala clustered with a recent Singapore isolate GU370053 collected in 2007. All the isolates obtained during 2011 confined to the lineage IV. 

### Recombination and selection pressure analysis

A data set comprising of 27 sequences [22 from the current study, NC_001475 reference sequence, GWL-25 (AY770511), ND143 (FJ644564) DEL-72 (GQ466079) and the Singapore isolate (GU370053)] was subjected to recombination analysis and selection pressure analysis. In the Datamonkey server used for the recombination analysis, both the SBP and GARD methods failed to detect any recombination events in the data set. Also, none among the eight methods implemented through the recombination detection program RDP3Beta34 identified recombination events. Nevertheless, in the selection pressure analysis, four among the five methods used identified various sites with statistically significant positive selection. The method SLAC failed to identify sites with positive selection. However, only the codon for I81 in the envelope protein was identified to be under positive selection by more than one method. Three programs- FEL (p-value 0.068), REL (Bayes factor 100.6) and FUBAR (posterior probability 0.9572) - identified this site. One strain RGCB837 had this site conserved with the reference sequence whereas three strains- RGCB350, RGCB429, RGCB632 had an alanine substitution, and the remaining 18 strains from the study showed a valine substitution (Figure 3).

## Discussion

Evolution through adaptive genetic changes plays a major role in making dengue virus a successful pathogen of the tropics [1,2]. It is fairly well established that such acquired changes affect the dengue disease severity [24,27]. Coupled with this, introduction of newer viral strains by cross-border transmission causes unprecedented changes in dengue disease spectrum in a region [13,28]. A keen monitoring of these events would be a major requirement for successful dengue surveillance and control programs.

Dengue virus has a uniform substitution rate across the structural and non-structural proteins. This permits the use of practically any of the genomic segments for evolutionary analysis [6,7]. Here, we selected the C-PrM-Envelope region. This region, being a structural protein, accumulates adaptive mutations under selection pressure for favoring infection of both human as well as mosquito cells. It also represents a sequence stretch that has extensively been used in previous studies [7,18]. Comparative sequence analysis of the 22 strains and other closely related strains revealed several mutations in the Kerala strains. Most of these mutations were genotype specific (Figure 3). Previous studies have shown that many adaptive mutations in the dengue viral genome map to the hinge region between the Domain I and II of the envelope protein [29], which is thought to be involved in the fusion with the cell membrane [30]. In our samples, no substitutions were observed in this region. Two asparagine residues (N67 and N153) in the E protein forming the N-linked glycosylation sites are considered important in interacting with the putative viral receptor [31,32]. Both of them were conserved in all the strains studied. Among the amino acid substitutions identified, the T219A mutation was a consistent change that fell within the Domain II of the envelope protein and was observed in 10 of the isolates studied (Figure 3). This change was later on identified as a signature substitution of the lineage IV strains as identified by the phylogenetic analysis (Figure 4).

DENV serotype 3 has five genotypes, and geographic confinement and independent evolution of these genotypes, especially the genotype I, II and III, have been noted earlier [33]. In fact, the Indian sub-continent was considered as the place of origin of the genotype III strains [24], with the virus spreading from Sri Lanka to the rest of the world [33]. Within DENV 3 genotype III, five lineages have been designated [10]. The earlier genotype III DENV-3 strains from India isolated in 1984 belonged to the lineage V, which was later replaced with lineage III strains that continues to circulate in the region [10,20]. This change was suggested as a cause for the increased incidence of severe dengue cases post-1990 [10]. In the present study we observe a shift from lineage III to lineage IV (Figure 4). The clade replacements leading to lineage shifts have resulted in the emergence of viral strains causing more severe forms of the disease in countries such as Sri Lanka [24] and Singapore [26]. In the former study, it was hypothesized that this distinction in disease severity may not be attributable to virus virulence alone, but could also be due to the dengue immunity background of the population. A pre-existing immunity to other serotypes can lead to preferential neutralization of virus from one lineage and at the same time, immune enhancement of infection of other lineage [24]. Taking clue from this, we verified the pre-existing immunity in the acute-phase samples from our patients by checking the presence of anti-dengue IgG and linked it with disease severity. Though 11 samples were IgG positive indicating secondary infections, they all represented uncomplicated dengue fever cases. So, it seems that the lineage shift *per se* has not resulted in a dramatic change in disease profile. DENV-1 and DENV-3 being the common serotypes in Kerala, one possibility could be that the existing background immunity against DENV-1 does not support a significant immune enhancement with these newer strains of DENV-3. Moreover, since the lineage shift seems to be of a recent occurrence, analysis of more number of clinical cases over a longer period might be essential to understand its true significance.

The close similarity of the lineage IV DENV-3 strains from the study to a Singapore strain is note-worthy. Among the three sites chosen for sample collection, two sites (Ernakulam and Thiruvananthapuram) have international airports, and all the lineage IV strains identified were from these locations. Interestingly, these strains were also genetically more divergent from this Singapore strain in the phylogenetic analysis indicating that they have undergone local evolution since their introduction into the country. Our study thus indicates that there is an inflow of DENV strains into the subcontinent along with the outward spread of the strains as identified earlier [33]. The presence of lineage III strains was confined to the third sample collection site (Pathanapuram). This place shared an interstate highway with the neighboring state of Tamilnadu from where dengue outbreak involving the north Indian GWL-25 strains were being reported [17].

As in many arboviruses, a strong purifying selection predominate the DENV evolution [12,34]. The occurrence of adaptive mutations in viral strains are dictated by the need for the virus to systematically replicate in the heterologous host systems –the man and the mosquito–which in turn has pointed to the incidence of convergent evolution via positive selection [12]. This is exemplified by the evidence of positive selection in the viral envelope protein residues in previous studies [8,35-38]. Envelope is the major antigenic protein of the dengue virus that is subjected to constant immune pressure. The I81 residue in the envelope protein that is identified to be under positive selection in our study (Figure 3) is located within the domain II of the protein. This is a region important in conformational change during low pH induced fusion of the virus with cell membrane [39,40]. Also, this residue is located in the solvent exposed loops in the tertiary structure of the envelope protein [37]. The changes in the I81 site, thus, can have functional consequences; however, the alanine / valine substitutions observed in our samples are conservative changes that might not affect protein secondary structure.

## Conclusion

The present study strengthens the earlier reports on the changing molecular pattern of dengue in India. Apart from sporadic genetic changes in the viral strains the study reveals genetic alterations resulting in lineage turn over. The lineage turnover is considered as a common phenomenon in dengue virus evolutionary dynamics, and such events in a population are followed by period of low prevalence of the new lineage strains [41]. These stochastic changes in one strain make way for the strains of other serotypes to flourish, thereby altering the disease epidemiology [41]. A temporal analysis of the dengue disease profile in the region is imperative to understand the long term sequel of the present observations. Coupled with a complete genome analysis of viral strains, this will enlighten our perception of the progression of dengue in the sub-continent, and support our efforts for its effective control.

## Materials and methods

### Clinical samples, RT-PCR-based virus detection and serology

An institution-based study involving three hospitals-the SAT Hospital, Medical College, Thiruvananthapuram; Community Health Centre, Pathanapuram, Kollam and the General Hospital, Ernakulam- was carried out. The first two hospitals are located in the two Southern most districts of Kerala, approximately 75 km apart; whereas the third one is located in central Kerala, approximately 250 km from Thiruvananthapuram (Figure 1). Acute-phase blood samples from dengue suspected febrile patients were obtained from the out-patient and in-patient departments of these hospitals during the period 2008–2011. Samples were obtained under informed consent, and un-linked samples were processed as per the Institutional Bio-safety Committee (IBSC) and Institutional Human Ethics committee (IHEC) approved protocols. Dengue viral RNA detection was done by an in-house RT-PCR that amplifies a 654 bp Core-Pre-membrane (C-PrM) coding region (nt from 132 to 783), as described previously [18,19]. Briefly, viral RNA was isolated from 140 μl of patient serum using QiAmp Viral RNA isolation system (Qiagen, Germany) and 5 μl of the RNA was RT-PCR amplified using single step RT-PCR kit (USB, Cleveland, Ohio) using D1F and DencomR2 primers for initial virus detection, or using D1F and nTS1/nTS2/nTS3/nDen4 primers (Table 2) for detection of the serotype. Presence of anti-dengue IgG antibodies in the RT-PCR positive patient serum samples was detected using a commercial ELISA kit (IVD Research Inc, Carlsbad, USA) as per the protocol supplied along with the kit. 

**Table 2:** Primers used in the study

**Primer name**	**Sequence (5’→ 3’)**	**Location (with respect to the ref. sequence NC_001475)**	**Reference sequence (GenBank ac. no)**	**Amplicon size**
D1F	TCAATATGCTGAACGCGCGAGAAACCG	132-159	NC_001477	
DencomR2	GCNCCTTCDGMNGACATCC	783-765	NC_001477	654 bp
nTS1	CTGGTTCCGTCTCAGTGATCCGGGGG	620-595	NC_001477	489 bp
nTS2	AACGCCACAAGGGCCATGAACA	254-233	AY858096	123 bp
nTS3	TGCTGGTAACATCATCATGAGACAGAGCG	427-399	NC_001475	296 bp
nDen4	CTCTGTTGTCTTAAACAAGAGAGGTC	527-502	NC_002640	395 bp
DV3EDIIIR	AAGCTTCTACTACTCGAACATCTTCCCAAT	2137-2120	NC_001475	2006 bp

### Infection of C6/36 cells and DENV detection by immunofluorescence

Dengue virus from RT-PCR positive samples was isolated in C6/36 *Aedes aegypti* cell line [18]. The cells were grown in L-15 medium with 10% Foetal Bovine serum (FBS; Invitrogen) and 1× antibiotic-antimycotic solution (Sigma) at 28°C. For infection, culture medium was removed from confluent cultures in 25 cm^2^ flasks (T-25) and an 1:10 dilution of patient serum in 1 ml culture medium was added. Flasks were incubated for 2 hrs at 28°C. Subsequently, the cells were washed twice with phosphate buffered saline (PBS; pH 7.4) and 5 ml of L-15 medium (Invitrogen, Carlsbad) with 2% FBS and 1× antibiotic-antimycotic solution (Sigma) was added. The cultures were incubated at 28°C for 5 days and the virus containing supernatants were collected and stored at −80°C in aliquots.

For immunofluorescence analysis, C6/36 cells were cultured as described above on glass cover slips placed in 24- well plates and infected as above with 200 μl of the 1:10 diluted virus sample. Cells were incubated at 28°C for three days. Medium was carefully removed from infected and uninfected control cells, and the cells were fixed with 4 % paraformaldehyde for 15 min at 4°C and washed 3 times with PBS. Permeabilization was done with 0.2% Triton-X 100 for 10 min at room-temperature and blocking was done with 8% normal goat serum (Sigma) in PBS for 1 hour at 37°C in a moist chamber. Primary antibody (Rabbit anti-dengue polyclonal serum; Catalogue No.GTX-29200; GeneTex;1:100 dilution) that can detect all the four serotypes of the virus was added and was incubated at 37°C for 1 hour in a moist-chamber. The cells were subsequently washed 3 times with PBS containing 0.1% Tween (PBS-T) and 1:500 dilution of goat anti-rabbit IgG Alexa Fluor®488 conjugated antibody (Cat. No.A11008, Molecular Probes, Invitrogen) was added and incubated for 30 min at room temperature. Cells were washed with PBS-T and then stained with DAPI (1 μg/ml). The cover slips were carefully mounted over the microscopic slides and were imaged using a Nikon TiS inverted fluorescent microscope. Images were captured with a LucaR (Andor) EMCCD camera using NIS elements software under identical exposure (500 milliseconds) and gain settings (6 units) for the infected cells as well as the controls. Cells treated only with the secondary antibody served as background fluorescent staining control in the experiments.

### Nucleotide sequencing and analysis

RNA from virus isolates obtained after a single passage in C6/36 cells was used for the RT-PCR amplification and nucleotide sequencing. Viral RNA was isolated from 140 μl of the C6/36 culture supernatant as described above, and was RT-PCR amplified using a high fidelity RT-PCR system (Fidelitaq RT-PCR kit; USB, Cleveland, Ohio) for amplifying a 2006 bp region spanning the C-PrM-Envelope coding regions. The RT-PCR reaction contained 10 pmol of the forward and reverse primers (D1F & DV3EDIII R; Table 2) and 5 μl of viral RNA in a 50 μl reaction. The amplification conditions were as described previously [18,19], except for an increased time for the extension step (2min). The amplified product was purified using the illustra GFX PCR purification Kit (GE Healthcare, Buckinghamshire, UK). Both strands of the amplicon were then sequenced with specific forward and reverse primers (Table 2) using the Big-dye Terminator Cycle sequencing kit in an ABI 3730 Genetic Analyzer automated DNA sequencer (PE Applied Biosystems, Foster City, CA). The comparative analysis of nucleotide and amino acid sequences of Indian as well as closely related Indo-Pacific isolates was carried out using the Clustal W function of the Bio-Edit 6.0.7software [42].

### Phylogenetic analysis

MEGA5.5 program [43] was used for phylogenetic analysis. The sequences used in the previous studies employing Indian strains [10] and the Indo-Pacific strains [8,33], and also the unpublished sequences of DENV-3 strains from the South and South East Asia and from the South America accessible from National Center for Biotechnology Information (NCBI) GenBank database (http://www.ncbi.nlm.nih.gov) were used as the dataset. The sequences were initially aligned by Clustal W [42] and the nucleotide substitution model for each data set was identified by the Model (ML) function in the MEGA5.5 program. Subsequently, maximum-likelihood analysis with 1000 boot-strap replications was carried out for the sequence data set using the identified substitution model.

### Recombination and selection pressure analysis

To detect recombination and identify recombination break points in the data alignment, two methods- SBP (Single Break Point recombination) and GARD (Genetic Algorithms for Recombination Detection) [44]- were initially carried out using the online adaptive evolution server http://www.datamonkey.org/[45]. Subsequently, a second program, RDP3Beta34 [46], was also used to reconfirm the results obtained. Default setting of this program for linear sequences was executed for implementing RDP, GENCONV [47], MAXCHI [48]; CHIMAERA [49]; BOOTSCAN [50]; SISCAN [51], 3SEQ [52] and PHYLPRO [53] in the analysis.

Selection pressure analysis was carried out in the server http://www.datamonkey.org/[45]. For identifying the residues with positive selection based on the ratio of non-synonymous to synonymous substitutions (dN/dS ratio), three different codon-based likelihood methods- Single Likelihood Ancestor counting (SLAC), Fixed Effects Likelihood (FEL) and Random Effects Likelihood (REL) - executed by the server were employed. The estimation of site-by-site substitution rates by the SLAC and FEL employs conservative methods whereas a less conservative method is used in the REL, which is best suited for small data sets with low sequence divergence. In addition, two newly introduced algorithms in the Datamonkey server -FUBAR and Mixed effects model evolution (MEME) were also executed to analyze the data set. Default parameters that used a Neighbour Joining tree and a significance setting of *P*-value/Bayes factor <0.1 for SLAC, FEL and MEME; a Bayes Factor of 50 for REL; and a posterior probability of 0.9 for FUBAR, were set in the analysis.

## Competing interests

The authors declare that they have no competing interests.

## Authors’ contributions

AM carried out RT-PCR, virus isolation and sequencing studies and drafted the manuscript. IJ, RPR, IR, LK was involved in clinical diagnosis of DENV patients and collecting blood samples. ES conceived the study, made the arrangements for obtaining samples from the hospitals and health centers, and finalized the manuscript. All authors read and approved the final manuscript.
